# Alcohol Use and HIV Suppression After Release From Prison Among People With HIV in Zambia

**DOI:** 10.1001/jamanetworkopen.2025.47295

**Published:** 2025-12-05

**Authors:** Michael E. Herce, Helene J. Smith, Vivien Mai, Chilambwe Mwila, Mirriam Nanyangwe, Tina Kayumba, Sisa Hatwiinda, Clement Moonga, Stephanie M. Topp, Izukanji Sikazwe, Jake M. Pry, Monde Muyoyeta

**Affiliations:** 1 Centre for Infectious Disease Research in Zambia, Lusaka, Zambia; 2 Institute for Global Health & Infectious Diseases, University of North Carolina at Chapel Hill, Chapel Hill; 3 School of Population Health, University of New South Wales, Sydney, Australia; 4 College of Medicine and Dentistry, James Cook University, Townsville, Australia; 5 Division of Epidemiology, University of California, Davis

## Abstract

**Importance:**

Little is known about the factors affecting viral suppression among people living with HIV returning to the community after incarceration (ie, reentrants) in Africa.

**Objective:**

To describe changes in viral suppression among reentrants living with HIV in Zambia and estimate the association between postrelease alcohol use and viral suppression.

**Design, Setting, and Participants:**

This cohort study enrolled incarcerated people living with HIV from 5 prisons and surrounding communities in Zambia and conducted study visits before and after prison release to assess HIV-associated health behaviors and changes in clinical outcomes between March 2017 and November 2019. Eligible participants were incarcerated people aged at least 18 years, living with HIV, with release planned fewer than 30 days from screening, enrolled in the national HIV program, planning on living in Lusaka postrelease, and providing voluntary informed consent. Data were analyzed from April 20, 2024, to October 2, 2025.

**Exposure:**

Postrelease unhealthy alcohol use (UAU), per the World Health Organization Alcohol Use Disorders Identification Test.

**Main Outcomes and Measures:**

The primary outcome was HIV-1 viral load (VL) suppression (<1000 copies/mL) before and after release. Mixed-effects Poisson regression was used to estimate the association between postrelease UAU and loss of viral suppression, using a directed acyclic graph to identify confounders.

**Results:**

Of 396 individuals screened, a total of 295 incarcerated people living with HIV (74.7%; median [IQR] age, 34 [29-41] years; 237 [80.3%] male) were included, with median (IQR) baseline CD4^+^ of 378 (244.5-506.5) cells/mL. At release, participants had been incarcerated a median (IQR) of 7.4 (3.5-15.4) months and receiving antiretroviral therapy for 13.2 (4.3-62.5) months. Documented viral suppression decreased from 237 individuals (80.3%) before release to 211 individuals (71.5%) after release. A total of 251 participants (85.1%) completed follow-up at a median (IQR) of 7.8 (5.4-11.6) months after release. UAU (prevalence ratio [PR], 3.35; 95% CI, 1.82-6.15) and unhealthy drug use (PR, 2.82; 95% CI, 1.39-5.71) were associated with postrelease unsuppressed VL. Among 205 participants (69.5%) with prerelease viral suppression, postrelease UAU was significantly associated with loss of viral suppression (adjusted risk ratio, 4.07; 95% CI, 1.97-8.42).

**Conclusions and Relevance:**

This cohort study of reentrants living with HIV in Zambia found that postrelease UAU was significantly associated with loss of viral suppression after community reentry. These findings suggest that new care models are needed to support HIV care continuity and address comorbid substance use in this population.

## Key Points

**Question:**

What is the association between unhealthy alcohol use and viral suppression among people living with HIV reentering the community after incarceration in Zambia?

**Findings:**

In this cohort study following 295 people living with HIV after prison release, postrelease unhealthy alcohol use was significantly associated with having an unsuppressed viral load and losing viral suppression (among those with suppressed viral load prerelease) at follow-up.

**Meaning:**

These findings suggest new care models are needed to address both substance use and HIV care continuity in this population.

## Teaser

This cohort study examines changes in viral suppression among people living with HIV after release from prison in Zambia and estimates the association between postrelease alcohol use and viral suppression.

## Introduction

In southern Africa, prisons concentrate large numbers of people living with HIV.^1,2^ In Zambia, more than 25 000 people are incarcerated at any given time.^3^ Estimates from Zambia indicate that HIV prevalence is several-fold higher among incarcerated people (14.3%-27.4%) than the adult general population (9.4%).^2,4,5,6,7,8^ Mounting evidence from Zambia and the region suggests that HIV treatment can be provided to people living with HIV in prisons, resulting in clinical benefits and viral suppression comparable to that seen in the community.^9,10,11^ Unfortunately, such benefits are thought to be short-lived for people living with HIV after prison release (ie, reentrants) due to fragmented HIV care.^2,12,13^ Based on scarce reports from Africa, one-third or more of reentrants are thought to not link to community HIV care or to experience HIV treatment interruption postrelease.^14,15^ Data from the Global North indicate that the first 6 months postrelease is a particularly high-risk period for poor clinical outcomes and HIV care disengagement for reentrants.^16,17,18,19,20,21,22^

Multiple psychosocial, structural, and health system barriers hinder HIV care for reentrants in Africa.^2,23^ Substance use disorders (SUDs), including alcohol use disorder (AUD) and drug use disorder (DUD), are thought to feature prominently.^20,21,22,23,24^ AUD, which is maladaptive alcohol use leading to harm or distress, is particularly relevant, as it is highly prevalent among both people who are incarcerated and people living with HIV in Africa.^25,26,27^ SUDs are also known to accelerate HIV disease progression and undermine antiretroviral therapy (ART) adherence.^28,29,30,31^ Despite high AUD prevalence among incarcerated populations globally, to our knowledge, no study from Africa has examined the association between unhealthy alcohol use (UAU), which encompasses the spectrum from alcohol consumption putting people at risk for but not yet causing harm (ie, hazardous alcohol use) to more clinically significant involvement (ie, severe AUD),^32,33,34^ and postrelease HIV clinical outcomes.^2^ Greater understanding of the association between UAU and HIV outcomes among reentrants is urgently needed, as UAU may lead to undue morbidity and mortality in this population^35^ and may contribute to HIV transmission in the communities to which reentrants return.

In this study, we aimed to describe HIV clinical outcomes and their associations with postrelease substance use among people living with HIV returning to the community from prison in Zambia. We hypothesized that postrelease UAU would be associated with loss of viral suppression for people living with HIV stably receiving treatment prior to prison release. In this report, we present new data that elucidate this association in Africa.

## Methods

This cohort study was approved by institutional review boards of the University of Zambia, Ministry of Health, University of North Carolina, James Cook University, and the US Office of Human Research Protections. All participants provided written informed consent. We report findings according to the Strengthening the Reporting of Observational Studies in Epidemiology (STROBE) reporting guideline for cohort studies.

### Study Design and Setting

We conducted a prospective cohort study in 5 correctional centers and surrounding communities (sites) in the Lusaka and Central Provinces, Zambia. These centers reflected diverse Zambia Correctional Service settings housing persons who have been sentenced and those on remand and awaiting trial, including 1 central prison, 1 remand facility, and 3 correctional complexes with maximum-, medium-, and minimal-security or open-air facilities. On-site and adjoining ART clinics provided HIV care, including HIV testing services and ART, according to national guidelines, which have been described previously.^23,36^

### Population

We enrolled a consecutive sample of adults (age ≥18 years) involved with the criminal justice system. Eligible participants were those who were documented to be living with HIV, incarcerated at a study site, scheduled for release within 30 days, enrolled in the national HIV treatment program, planning to live in Lusaka after release, and willing and able to provide voluntary informed consent and locator information.

### Procedures

Between March 13, 2017, and December 31, 2018, we recruited, screened, and enrolled participants prior to their release. Experienced study staff sensitized the population about the study using drama performances and through engagement with incarcerated peer health educators.^6^ Study staff then approached, in person, for recruitment purposes, all consecutive persons who were incarcerated and scheduled for discharge within 30 days. Participants completed 1 baseline study visit within 30 days of release and 1 follow-up study visit scheduled for approximately 6 months after release. All study visits included viral load (VL) measurement and collection of clinical, sociodemographic, and psychosocial data, including information about substance use. We provided simple alcohol-relevant advice and brief counselling according to World Health Organization (WHO) recommendations for participants identified with UAU,^37^ and specialist referral for participants with suspected SUD. During enrollment, participants completed a locator form with contact details to facilitate study follow-up after release. All participants were provided with mobile phone airtime at release. At approximately 3, 30, and 90 days after release, study staff conducted a telephone check-in to confirm participants’ best contact phone number and address and to remind participants of their study follow-up appointment. Follow-up occurred in the community, with participants determining the time and location of their visit, typically in a private space within a trusted gathering place (eg, school) or the participant’s home. We collected blood samples for HIV-1 VL testing, which was conducted using the cobas 4800 or Amplicor HIV-1 platform (Roche Molecular Systems) at the Centre for Infectious Disease Research in Zambia Laboratory. Each participant’s national HIV electronic medical record (EMR) history was reviewed through the last day of study observation (November 30, 2019) for evidence of routine clinic visits and VL measurement. Exit from the cohort occurred on the last day of study observation or if the participant died, requested early withdrawal, or became lost to study follow-up.

#### Routine Release Procedures

At release, Ministry of Health staff provided participants with a referral letter addressed to their preferred ART clinic to support linkage to community HIV care. Participants in treatment were also provided with a 30- or 60-day supply of ART and a smart card containing their digitized health record.^38^ All participants were counselled to present to their preferred ART clinic within 30 days of release to continue routine HIV care.

#### Independent Variables

The primary exposure of interest was presence of postrelease UAU, as defined by the WHO Alcohol Use Disorders Identification Test (AUDIT) over the period since release.^37^ The AUDIT’s excellent discriminatory ability and internal consistency for identification of UAU, including AUD, among people living with HIV in Zambia has been reported previously.^39^ AUDIT scores range 0 to 40, with scores of 8 or greater indicating UAU.^37^ Due to limited time allowed with incarcerated participants, we assessed alcohol use during and prior to incarceration with the abridged AUDIT-C.^40,41^ Given the possibility of comorbid DUD, we concurrently administered the Drug Use Disorders Identification Test (DUDIT).^42,43,44^ DUDIT scores range 0 to 44, with scores of 6 or greater and of 2 or greater suggestive of unhealthy drug use (UDU) in men and women, respectively.^42^ At the time of protocol development, DUDIT was the only drug use screening instrument validated in a justice-involved population.^45^ Covariates of interest included age, sex, marital status, incarceration status (ie, sentenced vs awaiting trial), incarceration duration, time since HIV diagnosis, time receiving ART, baseline WHO stage, baseline CD4^+^ count, and prerelease VL.

#### Outcomes

The primary outcome was postrelease HIV-1 viral suppression (ie, VL <1000 copies/mL). We also reported vital status in the national HIV treatment program (ie, retained in care, lost to follow-up, and died). We defined retained in care as at least 1 reported postrelease clinical encounter within 6 months of release or, for those who missed study follow-up, any documented encounter in the EMR prior to the last day of study observation without documented death.

#### Data Sources

We used case reporting forms, including a psychosocial survey containing the AUDIT-C, AUDIT, and DUDIT, to ascertain our primary exposure and covariates. All psychometric instruments were adapted to the Zambian correctional setting using WHO-recommended methods, with a focus on ensuring contextually relevant terms.^46^ We abstracted information from the national HIV EMR on all prerelease and postrelease clinical encounters, including data on ART dispensations, VL, and program vital status.

#### Study Size

We performed a sample size calculation a priori. We estimated that enrolling 386 participants would give us 82% power with a 2-sided α = .05 to detect a 15% absolute difference in our primary outcome between those with and without postrelease UAU.

#### Bias

Our primary analysis examined the association between postrelease UAU (exposure of interest) and postrelease viral suppression (outcome of interest) among participants with suppressed VL prior to release. We constructed a directed acyclic graph (DAG) to identify confounding variables for the primary analysis. The DAG was developed a priori based on prior research and investigator knowledge, according to modern methods, using the R package DAGitty (R Project for Statistical Computing) (eFigure 1 in Supplement 1).^47,48^ Using our DAG, we outlined the minimal adjustment set of confounding variables necessary to close all open back-door paths biasing estimation of the association between our exposure and outcome of interest (eFigure 1 in Supplement 1). Using Poisson regression, we estimated unadjusted and adjusted risk ratios (aRRs) with accompanying 95% CIs. The adjustment set for the adjusted model included age, sex, marital status, time incarcerated, preincarceration alcohol use, and time to postrelease VL measure (as a random effect). To interrogate the robustness of our findings, we conducted a sensitivity analysis using inverse probability weighting to account for data missingness due to those lost to study follow-up. We also completed a second sensitivity analysis to assess whether the observed associations held at an undetectable VL (ie, <60 copies/mL, the lower limit of detection for the study assay)^49^ to examine bias that may have occurred from different definitions for viral suppression.

### Statistical Analysis

For all study participants, we calculated summary statistics for variables of interest, reporting median and IQR for continuous variables and frequency and percentage for categorical variables. We compared binary outcomes using χ^2^ or Fisher exact test. We performed an exploratory analysis using Poisson regression to identify risk factors associated with unsuppressed viremia at study follow up and reported unadjusted prevalence ratios (PRs) with accompanying 95% CIs. All statistical analyses were performed using Stata 18 (StataCorp). Data were analyzed from April 20, 2024, to October 2, 2025.

## Results

### Participant Flow and Baseline Characteristics

We screened 396 incarcerated people and enrolled 296 individuals (74.7%), with 1 participant (0.3%) requesting study exit (Figure). Most participants were male (237 participants [80.3%]), and median (IQR) age was 34 (29-41) years. Participants had been incarcerated for a median (IQR) of 7.4 (3.5-15.4) months at enrollment (Table 1). At baseline, most participants had WHO stage I disease (164 participants [55.6%]), were virally suppressed (237 participants [80.3%]), and had a median (IQR) CD4^+^ of 378 (244.5-506.5) cells/mL. Participants had been receiving ART for a median of 402 (4.3-62.5) days (or 13.2 months) at release, typically treated with tenofovir disoproxil fumarate plus emtricitabine/lamivudine plus efavirenz. A total of 58 participants (19.7%) reported UAU in the 3 months preceding incarceration, with drug use preincarceration much less common (18 participants [6.1%]). No participant endorsed alcohol use while incarcerated.

**Figure.**
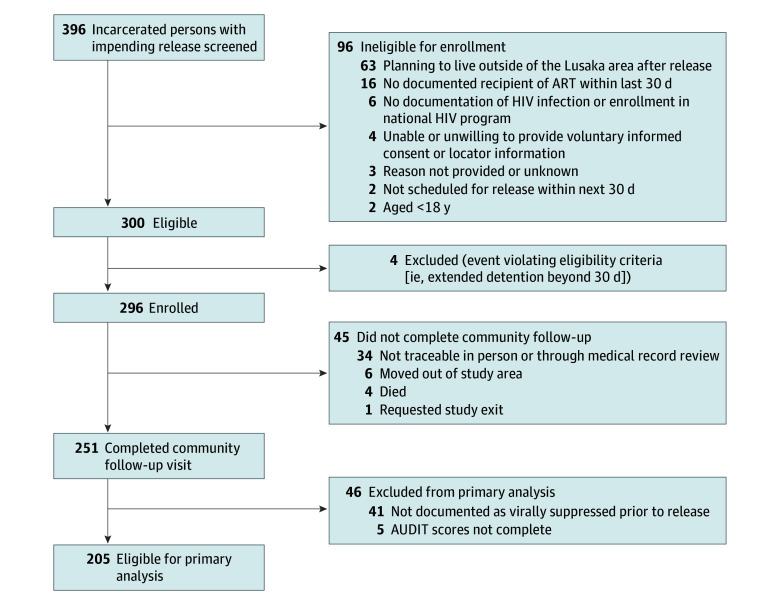
Participant Flow Through the Study AUDIT indicates Alcohol Use Disorders Identification Test.

**Table 1.** Baseline Characteristics for Participants in Total, Who Completed the 6-Month Postrelease Follow-Up Visit, and Who Were Included in the Primary Analysis

Factor	Participants, No. (%)		
	Total (N = 295)	Completed study follow-up (n = 251)	Primary analysis (n = 205)
Birth sex			
Male	237 (80.3)	203 (80.9)	169 (82.4)
Female	58 (19.7)	48 (19.1)	36 (17.6)
Age, y			
Median (IQR)	34 (29-41)	34 (29-41)	35 (20-42)
18-24	27 (9.2)	25 (10.0)	17 (8.3)
25-29	53 (18.0)	40 (15.9)	28 (13.7)
30-34	76 (25.8)	65 (25.9)	51 (24.9)
35-39	57 (19.3)	48 (19.1)	42 (20.5)
40-44	46 (15.6)	42 (16.7)	39 (19.0)
≥45	36 (12.2)	31 (12.4)	28 (13.7)
Marital status			
Married	169 (57.3)	146 (58.2)	121 (59.0)
Cohabitating	1 (0.3)	0	0
Widowed	24 (8.1)	21 (8.4)	17 (8.3)
Divorced or separated	53 (18.0)	43 (17.1)	34 (16.6)
Never married	47 (15.9)	40 (15.9)	32 (15.6)
Unknown	1 (0.3)	1 (0.4)	1 (0.5)
History of prior incarceration			
No	272 (92.2)	230 (91.6)	185 (90.2)
Yes	23 (7.8)	21 (8.4)	20 (9.8)
Duration of index incarceration			
Median (IQR), d	224 (107-468)	237 (115-494)	241 (120-560)
≤1 mo (30 d)	25 (8.5)	17 (6.8)	9 (4.4)
>1-6 mo	103 (34.9)	87 (34.7)	73 (35.6)
>6-24 mo	120 (40.7)	106 (42.2)	87 (42.4)
>24 mo	47 (15.9)	41 (16.3)	36 (17.6)
Incarceration status			
Sentenced	162 (54.9)	136 (54.2)	107 (52.2)
Awaiting trial	133 (45.1)	115 (45.8)	98 (47.8)
Time since HIV diagnosis			
Median (IQR), d	386 (114-1928)	429 (111-2073)	595 (124-2336)
≤1 mo (30 d)	26 (8.8)	21 (8.4)	9 (4.4)
>1-6 mo	76 (25.8)	67 (26.7)	56 (27.3)
>6-24 mo	72 (24.4)	59 (23.5)	45 (22.0)
WHO stage			
1	164 (55.6)	136 (54.2)	120 (58.5)
2	31 (10.5)	27 (10.8)	18 (8.8)
3	18 (6.1)	14 (5.6)	12 (5.9)
4	1 (0.3)	1 (0.4)	1 (0.5)
Missing	81 (27.5)	73 (29.1)	54 (26.3)
CD4^+^ cell count, cells/mm^3^			
Median (IQR)	378 (244.5-506.5)	386 (248-507)	398 (269-508)
<200	44 (14.9)	37 (14.7)	26 (12.7)
200-349	84 (28.5)	67 (26.7)	54 (26.3)
350-499	83 (28.1)	74 (29.5)	68 (33.2)
≥500	73 (24.7)	63 (25.1)	55 (26.8)
Missing	11 (3.7)	10 (4.0)	2 (1.0)
Time since ART initiation			
Median (IQR), d	402 (131-1899)	405.5 (119.5-1930)	522 (122-2202)
≤1 mo (30 d)	15 (5.1)	13 (5.2)	9 (4.4)
>1-6 mo	75 (25.4)	67 (26.7)	58 (28.3)
>6-24 mo	76 (25.8)	63 (25.1)	48 (23.4)
>24 mo	113 (38.3)	97 (38.6)	90 (43.9)
Missing	16 (5.4)	11 (4.4)	0 (0.0)
ART regimen			
FDC tenofovir disoproxil fumarate + emtricitabine/ lamivudine + efavirenz	275 (93.2)	236 (94.0)	194 (94.6)
Tenofovir disoproxil fumarate + emtricitabine/ lamivudine + nevirapine	2 (0.7)	1 (0.4)	1 (0.5)
Other	2 (0.7)	2 (0.8)	2 (1.0)
Missing	16 (5.4)	12 (4.8)	8 (3.9)
Prerelease viral suppression status^a^			
Suppressed	237 (80.3)	209 (83.3)	205 (100.0)
Unsuppressed	50 (16.9)	35 (13.9)	0
Missing	8 (2.7)	7 (2.8)	0
Preincarceration alcohol use^b^			
No unhealthy alcohol use	207 (70.2)	170 (67.7)	144 (70.2)
Unhealthy alcohol use	58 (19.7)	51 (20.3)	39 (19.0)
Missing	30 (10.2)	30 (12.0)	22 (10.7)
Preincarceration drug use^c^			
No unhealthy drug use	232 (78.6)	232 (92.4)	191 (93.2)
Unhealthy drug use	18 (6.1)	18 (7.2)	14 (6.8)
Missing	45 (15.3)	1 (0.4)	0

Abbreviations: ART, antiretroviral therapy; FDC, fixed-dose combination; WHO, World Health Organization.

^a^ Unsuppressed viral load was defined as 1000 copies/mL or greater.

^b^ As assessed by verbal interview using the Alcohol Use Disorders Identification Test–Consumption.

^c^ As assessed by verbal interview using the Drug Use Disorders Identification Test.

We ascertained vital status in the national HIV program for all participants 6 months after release: 254 participants (86.1%) were alive and retained in care, 37 participants (12.5%) were lost to follow-up, and 4 participants (1.4%) had died (Table 2). Among all participants, documented viral suppression decreased to 211 participants (71.5%) after release. Of all participants, 251 (85.1%) completed study follow-up, at a median (IQR) of 7.8 (5.4-11.6) months after release (eFigure 2 in Supplement 1), and were eligible for the exploratory viremia risk factor analysis. There were 44 participants (14.9%) who did not undergo study follow-up, accounting for the 44 (14.9%) participants missing a postrelease VL and most of the 51 participants (17.3%) missing a postrelease AUDIT score.

**Table 2.** Participant Characteristics at Study Follow-Up

Factor	Participants, No. (%)		
	Total (N = 295)	Completed study follow-up (n = 251)	Primary analysis (n = 205)
Vital status in the national HIV program			
Alive and retained in care	254 (86.1)	251 (100)	205 (100)
Lost to program follow-up	37 (12.5)	0	0
Died	4 (1.4)	0	0
Postincarceration alcohol use^a^			
No unhealthy alcohol use	226 (76.6)	226 (90.0)	190 (92.7)
Unhealthy alcohol use	18 (6.1)	18 (7.2)	15 (7.3)
Missing	51 (17.3)	7 (2.8)	0
Postincarcetation drug use^b^			
No unhealthy drug use	232 (78.6)	232 (92.4)	191 (93.2)
Unhealthy drug use	18 (6.1)	18 (7.2)	14 (6.8)
Missing	45 (15.3)	1 (0.4)	0 (0.0)
Postrelease viral suppression status^c^			
Suppressed	211 (71.5)	211 (84.1)	180 (87.8)
Unsuppressed	40 (13.6)	40 (15.9)	25 (12.2)
Missing	44 (14.9)	0	0

^a^ As assessed by verbal interview using the Alcohol Use Disorders Identification Test.

^b^ As assessed by verbal interview using the Drug Use Disorders Identification Test.

^c^ Unsuppressed viral load was defined as 1000 copies/mL or greater.

Of 251 participants who completed study follow-up, 18 (7.2%) reported UAU and 18 (7.2%) reported UDU (Table 2). In total, 30 participants (12.0%) reported behaviors consistent with UAU or UDU, and 6 participants (2.4%) reported overlapping UAU and UDU. We calculated Cronbach α for AUDIT and DUDIT responses at 0.79 and 0.92, respectively, indicating good to excellent internal consistency. In exploratory analyses, having an unsuppressed VL at follow up was associated with UAU (PR, 3.35; 95% CI, 1.82-6.15; *P* < .001) and UDU (PR, 2.82; 95% CI, 1.39-5.71; *P* = .004) after release (Table 3). Having an unsuppressed VL after release was also significantly associated with being younger (age 25-29 vs 35-29 years: PR, 4.20; 95% CI, 1.48-11.91; *P* = .007), having been receiving ART for 6 to 24 months (vs >24 months: PR, 2.40; 95% CI,1.18-4.90; *P* = .02), and having an unsuppressed VL before release (PR, 3.11; 95% CI, 1.78-5.43; *P* < .001).

**Table 3.** Exploratory Risk Factor Analysis for Unsuppressed Viral Load Postrelease (n = 251)^a^

Factor	PR (95% CI)	*P* value
Unhealthy alcohol use^b^		
No	1 [Reference]	NA
Yes	3.35 (1.82-6.15)	<.001
Unhealthy drug use^c^		
No	1 [Reference]	NA
Yes	2.82 (1.39-5.71)	.004
Age, y		
18-24	1.44 (0.35-5.95)	.62
25-29	4.20 (1.48-11.91)	.007
30-34	2.40 (0.83-6.94)	.11
35-39	1 [Reference]	NA
40-44	1.43 (0.40-5.09)	.58
≥45	0.39 (0.04-3.33)	.39
Sex		
Male	1 [Reference]	NA
Female	1.23 (0.62-2.45)	.56
Marital status		
Married	1 [Reference]	NA
Widowed	1.16 (0.44-3.02)	.76
Divorced/separated	0.85 (0.36-1.98)	.71
Never married	0.91 (0.40-2.08)	.83
Time incarcerated, mo		
≤1	1.45 (0.39-5.37)	.58
>1-6	1.41 (0.54-3.69)	.48
>6-24	1.32 (0.51-3.38)	.57
>24	1 [Reference]	NA
Incarceration status		
Sentenced	1 [Reference]	NA
Awaiting trial	0.97 (0.54-1.72)	.91
Previously incarcerated		
No	1 [Reference]	NA
Yes	0.58 (0.15-2.24)	.43
Time since ART initiation, mo		
≤1	1.35 (0.41-4.45)	.62
>1-6	1.55 (0.71-3.39)	.27
>6-24	2.40 (1.18-4.9)	.02
>24	1 [Reference]	NA
Prerelease viral load^a^		
Suppressed	1 [Reference]	NA
Unsuppressed	3.11 (1.78-5.43)	<.001

Abbreviations: ART, antiretroviral therapy; NA, not applicable; PR, prevalence ratio.

^a^ Unsuppressed viral load was defined as 1000 copies/mL or greater.

^b^ As assessed by verbal interview using the Alcohol Use Disorders Identification Test.

^c^ As assessed by verbal interview using the Drug Use Disorders Identification Test.

A subset of participants (205 of 295 participants [69.5%]) had suppressed VL before release and completed both follow-up VL testing and substance use assessment and were included in our primary analysis. These participants tended to have been receiving ART longer than the total study population (Table 1). Characteristics of individuals included and not included in the primary analysis are further detailed in eTable 1 in Supplement 1. In adjusted analyses, reentrants with UAU were significantly more likely to lose viral suppression after release compared with those reporting no UAU (adjusted RR, 4.07; 95% CI, 1.97-8.42; *P* < .001) (Table 4). In sensitivity analyses, this association remained statistically significant at a more stringent viral suppression threshold (eTable 2 in Supplement 1). Similarly, people with UDU were more likely to lose viral suppression on community reentry compared with those reporting no UDU (3.84; 95% CI, 1.43-10.30; *P* = .008). Using inverse probability weighting to account for those lost to study follow-up, UAU (aRR, 3.95; 95% CI, 1.12-13.90; *P* = .03) and UDU (aRR, 5.67; 95% CI, 1.72-18.68; *P* = .004) remained significantly associated with loss of viral suppression postrelease (eTable 3 in Supplement 1).

**Table 4.** Primary Analysis Estimating Risk of Loss of Viral Suppression Postrelease Among Participants With Viral Suppression Prerelease (N = 205)^a^

Factor	RR (95% CI)	*P* value	aRR (95% CI)^b^	*P* value
Unhealthy alcohol use^c^				
No	1 [Reference]	NA	1 [Reference]	NA
Yes	4.00 (1.90-8.42)	<.001	4.07 (1.97-8.42)	<.001
Age, y				
18-24	0.82 (0.09-7.40)	.86	0.83 (0.05-12.93)	.89
25-29	4.50 (1.32-15.33)	.02	4.97 (1.49-16.58)	.01
30-34	1.92 (0.53-6.98)	.32	2.10 (0.58-7.65)	.26
35-39	1 [Reference]	NA	1 [Reference]	NA
40-44	1.44 (0.33-6.20)	.63	1.67 (0.38-7.41)	.50
≥45	0.50 (0.05-4.61)	.54	0.45 (0.05-4.00)	.48
Sex				
Male	1 [Reference]	NA	1 [Reference]	NA
Female	0.89 (0.32-2.48)	.89	0.90 (0.28-2.93)	.86
Marital status				
Married	1 [Reference]	NA	1 [Reference]	NA
Widowed	1.02 (0.25-4.18)	.98	1.69 (0.38-7.50)	.49
Divorced/separated	1.02 (0.35-2.93)	.98	0.62 (0.22-1.74)	.37
Never married	1.35 (0.52-3.52)	.54	0.98 (0.31-3.11)	.97
Time incarcerated, mo				
≤1	8.00 (0.81-78.97)	.08	7.65 (0.76-77.28)	.09
>1-6	5.42 (0.71-41.72)	.10	4.89 (0.63-37.75)	.13
>6-24	4.55 (0.59-34.88)	.15	4.45 (0.56-35.14)	.16
>24	1 [Reference]	NA	1 [Reference]	NA
Preincarceration alcohol use^d^				
No unhealthy alcohol use	1 [Reference]	NA	1 [Reference]	NA
Unhealthy alcohol use	0.87 (0.31-2.43)	.79	0.69 (0.26-1.82)	.45
Missing	1.54 (0.57-4.18)	.40	1.58 (0.58-4.31)	.37

Abbreviations: aRR, adjusted risk ratio; NA, not applicable.

^a^ Unsuppressed viral load was defined as 1000 copies/mL or greater.

^b^ Adjustment set: age, sex, marital status, time incarcerated, pre-incarceration alcohol use, and time to post-release viral load measure (as random effect).

^c^ As assessed by verbal interview using the Alcohol Use Disorders Identification Test.

^d^ As assessed by verbal interview using the Alcohol Use Disorders Identification Test–Consumption.

## Discussion

In this cohort study, we prospectively followed people living with HIV before and after prison release and examined factors associated with unsuppressed viremia and loss of viral suppression on community reentry, contributing new and largely unavailable data for this population. We observed a decrease in the proportion of participants with documented viral suppression after release. Notably, we identified postrelease UAU and UDU as risk factors associated with unsuppressed VL and loss of prerelease viral suppression on return to the community. These findings point to potential behavioral and structural determinants of poor HIV clinical outcomes among justice-involved people living with HIV in Africa and suggest that new models of care are needed to both support HIV care engagement and address comorbid substance use in this key population.

In Zambia and elsewhere in Africa, incarcerated people are increasingly accessing ART, with several reports of favorable clinical outcomes being achieved in on-site and referral-based HIV treatment programs.^6,11,14,50^ In a universal test and treat program in Zambia and South Africa, viral suppression reached 97% for people living with HIV who remained incarcerated for at least 6 months following treatment initiation.^11^ In a large South African program, 1935 incarcerated people living with HIV were initiated to ART, and 898 individuals (79%) achieved viral suppression (defined as <400 copies/mL) among those with a documented result.^51^ Taken together, these data suggest that providing comprehensive HIV services to incarcerated people may create unique opportunities to reach a highly marginalized population with ART and its individual and public health benefits. However, comparatively little is known about whether these benefits can be sustained for people living with HIV in African settings after release from prison and how they may fare clinically in the face of multiple postrelease barriers to HIV care. These barriers may be felt most acutely by individuals who are detained while awaiting trial and often released directly (and unpredictably) from court.^23^ As a result, they may be less likely to receive recommended discharge planning services at release than sentenced people living with HIV, with potential for adverse health consequences.

In one of the few studies evaluating postrelease outcomes in Africa,^15^ 516 incarcerated people living with HIV from South Africa were followed-up after release, of whom 351 (68%) were released while receiving ART and had postrelease linkage to care assessed. In the study by Mabuto et al,^15^ 65% of participants self-reported linking to HIV care in the community, with only 34% verified in the medical record, leading the authors to conclude that at most 47% had no lapse in ART. In another small study from South Africa, only 23 of 34 released people living with HIV (68%) had at least 1 clinical visit in the community, suggesting interrupted HIV care after release.^14^ Neither of these studies measured HIV VL or assessed risk factors associated with uncontrolled viremia.

While relatively few participants in our study reported unhealthy substance use, we nonetheless observed a significant association between postrelease UAU and loss of viral suppression for participants whose VL was suppressed prerelease. This association held after controlling for confounding and in sensitivity analyses using a more stringent threshold for viral suppression and inverse probability weighting to adjust for loss to study follow-up. While we noted a similar association with UDU, UAU may be more relevant, as it is more prevalent in Zambia, where alcohol is more widely available than drugs.^40^ Our findings fit with other data on alcohol use among justice-involved persons in Africa, where upwards of 40% may have some form of AUD.^29^ While we documented the extent of substance use among formerly incarcerated people living with HIV returning to the community specifically in Zambia, AUD is increasingly being identified as a problem for formerly incarcerated populations throughout the region.^2^ Reports from Nigeria and South Africa have documented high AUD prevalence among incarcerated persons,^29,52,53^ with other studies documenting a lifetime AUD prevalence of 80% among individuals who have been incarcerated.^28,30^ These results reflect broader trends in Africa, where alcohol consumption is increasing^54^ and as many as 25% of men living with HIV may have alcohol dependence.^25,55^ Similarly, limited data from the region suggest that UDU is an issue, with approximately 5% of detainees reporting cannabis use in the past month in Nigeria^28^ and Kenya.^30^ Like with AUD, all SUDs can compromise HIV treatment and care for formerly incarcerated populations by undermining HIV care engagement and treatment adherence.^56,57,58^

Our findings suggest an urgent need to develop differentiated HIV care models to support people living with HIV reentering the community after prison release. Such models can incorporate 1 or more evidence-based interventions, such as case management,^59^ peer navigation,^60^ and group support,^61^ to improve linkage to community HIV care and treatment adherence. Second, any transitional care models must address comorbid UAU and UDU. While evidence-based approaches to address these conditions are lacking for this population in Africa, peer-based cognitive behavioral-based therapies could play a role.^62^ Other evidence-based interventions that could be adapted for this population and merit further research include brief counselling interventions,^63^ motivational interviewing,^64^ contingency management,^65^ and the community reinforcement approach.^33,66^

### Limitations

Although we successfully followed a highly mobile population seeking anonymity, our study has several limitations. First, we did not achieve our target sample size due to the logistical complexity of identifying and enrolling incarcerated persons before release. While this did not limit our ability to detect an association in our primary analysis, it may have restricted the precision of estimates. Critically, while 85% of participants successfully completed a study follow-up visit, some participants were lost due to their movement outside the study area and the substantial time and resources involved with conducting study procedures in the community. However, we attempted to account for study loss to follow-up in a dedicated sensitivity analysis, and the significance of the association between UAU and loss of viral suppression held. Third, contact between the study team and participants may have contributed to the higher retention in care that we observed compared with previous reports. Indeed, our team’s contact with participants may have functioned as a de facto HIV care retention intervention, given the paucity of transitional care supports available to this population.^2^ Based on these considerations, we conjecture that the results we observed likely represent a conservative best-case scenario for HIV clinical outcomes among reentrants in Zambia. Fourth, based on our qualitative results,^23^ we suspect that social desirability bias may have led to underreporting of UAU and UDU in the cohort. Fifth, although the DUDIT has been used in Africa previously^67^ and has shown favorable performance characteristics in an incarcerated population,^68^ its sensitivity and positive predictive value may be lower for identifying less severe UDU, limiting its utility for this condition.^44^

## Conclusions

This cohort study found that previously incarcerated reentrants living with HIV in Zambia faced substantial risk of treatment interruption resulting in loss of viral suppression associated with UAU and UDU in the period immediately following prison release. This observation has implications for policies and programs to support HIV care continuity for people living with HIV transitioning from prison to the community in Africa. To change the current reality for reentrants living with HIV, governments, donors, and implementing partners must meaningfully include justice-involved people in HIV programming, strengthen corrections health systems at their interface with community health systems, and empower civil society organizations to better serve the incarcerated and formerly incarcerated population in Africa.

## Notes

Supplement 1. eTable 1. Study population overall and by primary analysis status (N=295) eTable 2. Risk of loss of viral suppression (at a threshold of ≥60 copies/ml) among participants who were virally suppressed pre-release (N=205) eTable 3. Inverse probability weighted risk ratios for loss of viral suppression among participants with vs without unhealthy alcohol use or unhealthy drug use who were virally suppressed pre-release (N=205) eFigure 1. Directed acyclic graph (DAG), featuring minimal adjustment set of age, marital status, sex, pre- incarceration alcohol use, and time incarcerated eFigure 2. Histogram of time from release to study follow-up completion (N=251)

Supplement 2. Data Sharing Statement
