# Supplementary material for: Alcohol Use and HIV Suppression After Release From Prison Among People With HIV in Zambia
Source: JAMA Netw Open. 2025 Dec 5;8(12):e2547295. doi: 10.1001/jamanetworkopen.2025.47295 (PMC12681033; doi:10.1001/jamanetworkopen.2025.47295)
Supplement: Supplement 1. — eTable 1. Study population overall and by primary analysis status (N=295) eTable 2. Risk of loss of viral suppression (at a threshold of ≥60 copies/ml) among participants who were virally suppressed pre-release (N=205) eTable 3. Inverse probability weighted risk ratios for loss of viral suppression among participants with vs without unhealthy alcohol use or unhealthy drug use who were virally suppressed pre-release (N=205) eFigure 1. Directed acyclic graph (DAG), featuring minimal adjustment set of age, marital status, sex, pre- incarceration alcohol use, and time incarcerated eFigure 2. Histogram of time from release to study follow-up completion (N=251) [file jamanetwopen-e2547295-s001.pdf]

Supplemental Online Content

Herce ME, Smith HJ, Mai V, et al. Alcohol use and HIV suppression after release from prison among people with HIV in Zambia. *JAMA Netw Open*. 2025;8(12):e2547295.
doi:10.1001/jamanetworkopen.2025.47295

eTable 1. Study population overall and by primary analysis status (N=295)
eTable 2. Risk of loss of viral suppression (at a threshold of ≥60 copies/ml) among participants who were virally suppressed pre-release (N=205)
eTable 3. Inverse probability weighted risk ratios for loss of viral suppression among participants with vs without unhealthy alcohol use or unhealthy drug use who were virally suppressed pre-release (N=205)
eFigure 1. Directed acyclic graph (DAG), featuring minimal adjustment set of age, marital status, sex, pre-incarceration alcohol use, and time incarcerated
eFigure 2. Histogram of time from release to study follow-up completion (N=251)

This supplemental material has been provided by the authors to give readers additional information about their work.

© 2025 Herce ME et al. *JAMA Network Open*.

eTable 1. Study population overall and by primary analysis status (N=295).

Factor	Level	Total<br>n (%)	Primary Analysis<br>n (%)	Not in Primary Analysis<br>n (%)	p-value
N		295	205	90	
**Sex**	Male	237 (80.3)	169 (82.4)	68 (75.6)	0.17
	Female	58 (19.7)	36 (17.6)	22 (24.4)	
**Age**	18-24 years	27 (9.2)	17 (8.3)	10 (11.1)	0.01
	25-29 years	53 (18.0)	28 (13.7)	25 (27.8)	
	30-34 years	76 (25.8)	51 (24.9)	25 (27.8)	
	35-39 years	57 (19.3)	42 (20.5)	15 (16.7)	
	40-44 years	46 (15.6)	39 (19.0)	7 (7.8)	
	45+ years	36 (12.2)	28 (13.7)	8 (8.9)	
**Marital status**	Married	169 (57.3)	121 (59.0)	48 (53.3)	0.57
	Cohabitating	1 (0.3)	0 (0.0)	1 (1.1)	
	Widowed	24 (8.1)	17 (8.3)	7 (7.8)	
	Divorced/Separated	53 (18.0)	34 (16.6)	19 (21.1)	
	Never married	47 (15.9)	32 (15.6)	15 (16.7)	
	Unknown	1 (0.3)	1 (0.5)	0 (0.0)	
**CD4 cell count (cells/mm^3)**	median (IQR)	378 (244.5-506.5)	398 (269-508)	323 (213-465)	0.008
	<200	44 (14.9)	26 (12.7)	18 (20.0)	<0.001
	200-349	84 (28.5)	54 (26.3)	30 (33.3)	
	350-499	83 (28.1)	68 (33.2)	15 (16.7)	
	500+	73 (24.7)	55 (26.8)	18 (20.0)	
	Missing	11 (3.7)	2 (1.0)	9 (10.0)	
**WHO stage**	1	164 (55.6)	120 (58.5)	44 (48.9)	0.44
	2	31 (10.5)	18 (8.8)	13 (14.4)	
	3	18 (6.1)	12 (5.9)	6 (6.7)	
	4	1 (0.3)	1 (0.5)	0 (0.0)	
	Missing	81 (27.5)	54 (26.3)	27 (30.0)	
**Post-incarceration alcohol use[a]**	No unhealthy alcohol use	207 (70.2)	173 (84.4)	34 (37.8)	<0.001
	Unhealthy alcohol use	42 (14.2)	32 (15.6)	10 (11.1)	
	Missing	46 (15.6)	0 (0.0)	46 (51.1)	
**Pre-incarceration alcohol use[b]**	No unhealthy alcohol use	207 (70.2)	144 (70.2)	63 (70.0)	0.84
	Unhealthy alcohol use	58 (19.7)	39 (19.0)	19 (21.1)	
	Missing	30 (10.2)	22 (10.7)	8 (8.9)	
**Post-incarceration drug use[c]**	No unhealthy drug use	232 (78.6)	191 (93.2)	41 (45.6)	<0.001
	Unhealthy drug use	18 (6.1)	14 (6.8)	4 (4.4)	
	Missing	45 (15.3)	0 (0.0)	45 (50.0)	
**Time since HIV diagnosis**	median (IQR), in days	386 (114-1928)	595 (124-2336)	236.5 (62-864)	<0.001
	≤30 days	26 (8.8)	9 (4.4)	17 (18.9)	<0.001
	>1-6 months	76 (25.8)	56 (27.3)	20 (22.2)	
	>6-24 months	72 (24.4)	45 (22.0)	27 (30.0)	
	>25 months	121 (41.0)	95 (46.3)	26 (28.9)	
**Time on ART at enrollment**	Median (IQR), in days	402 (131-1899)	522 (122-2202)	281 (153-1039)	0.09
	≤1 month	15 (5.1)	9 (4.4)	6 (6.7)	<0.001

© 2025 Herce ME et al. *JAMA Network Open*.

	>1-6 months	75 (25.4)	58 (28.3)	17 (18.9)	
	>6-24 months	76 (25.8)	48 (23.4)	28 (31.1)	
	>24 months	113 (38.3)	90 (43.9)	23 (25.6)	
	Missing	16 (5.4)	0 (0.0)	16 (17.8)	
**ART regimen**	FDC tenofovir disoproxil fumarate + emtricitabine/ lamivudine + efavirenz	254 (86.1)	181 (88.3)	73 (81.1)	0.22
	FDC Tenofovir disoproxil fumarate + emtricitabine/ lamivudine + nevirapine	2 (0.7)	1 (0.5)	1 (1.1)	
	Other	2 (0.7)	2 (1.0)	0 (0.0)	
	Missing	37 (12.5)	21 (10.2)	16 (17.8)	
**Incarceration Status**	Sentenced	162 (54.9)	107 (52.2)	55 (61.1)	0.16
	Awaiting trial/ Remandee	133 (45.1)	98 (47.8)	35 (38.9)	
**Time incarcerated**	Median (IQR), in days	224 (107-468)	241 (120-560)	176.5 (49-314)	0.002
	≤1 month	25 (8.5)	9 (4.4)	16 (17.8)	0.002
	>1-6 months	103 (34.9)	73 (35.6)	30 (33.3)	
	>6-24 months	120 (40.7)	87 (42.4)	33 (36.7)	
	>24 months	47 (15.9)	36 (17.6)	11 (12.2)	
**Previously incarcerated**	No	272 (92.2)	185 (90.2)	87 (96.7)	0.06
	Yes	23 (7.8)	20 (9.8)	3 (3.3)	
**Pre-release viral suppression[d]**	Suppressed	237 (80.3)	205 (100.0)	32 (35.6)	<0.001
	Unsuppressed	50 (16.9)	0 (0.0)	50 (55.6)	
	Missing	8 (2.7)	0 (0.0)	8 (8.9)	
**Post-release viral suppression[d]**	Suppressed	211 (71.5)	180 (87.8)	31 (34.4)	<0.001
	Unsuppressed	40 (13.6)	25 (12.2)	15 (16.7)	
	Missing	44 (14.9)	0 (0.0)	44 (48.9)	

Note: IQR- interquartile range; WHO- World Health Organization; ART- antiretroviral therapy; FDC- Fixed-dose combination;
[a]As assessed by verbal interview using the AUDIT.
[b]As assessed by verbal interview using the AUDIT-C.
[c]As assessed by verbal interview using the DUDIT.
[d]Unsuppressed viral load was defined as 1000 copies/mL or greater

© 2025 Herce ME et al. *JAMA Network Open*.

eTable 2. Risk of loss of viral suppression (at a threshold of ≥60 copies/ml) among participants who were virally suppressed pre-release (N=205).

Factor	Level	Unadjusted		Adjusted	
		RR (95% CI)	p-value	aRR (95% CI)	p-value
**Unhealthy alcohol use[a]**	No	1.00 (ref)	-	1.00 (ref)	-
	Yes	2.98 (1.72, 5.15)	<0.001	2.82 (1.59, 4.99)	<0.001
**Unhealthy drug use[b]**	No	1.00 (ref)	-	1.00 (ref)	-
	Yes	2.73 (1.50, 4.96)	0.001	1.78 (0.82, 3.89)	0.15
**Age**	18-24 years	1.24 (0.32, 4.70)	0.76	1.53 (0.34, 6.99)	0.76
	25-29 years	2.75 (1.14, 6.65)	0.03	2.52 (1.04, 6.14)	0.03
	30-34 years	1.51 (0.61, 3.72)	0.37	1.62 (0.67, 3.94)	0.37
	35-39 years	1.00 (ref)	-	1.00 (ref)	-
	40-44 years	1.26 (0.45, 3.5)	0.66	1.33 (0.47, 3.72)	0.66
	45+ years	1.00 (0.32, 3.16)	1.00	1.05 (0.32, 3.44)	0.94
**Sex**	Male	1.00 (ref)	-	1.00 (ref)	-
	Female	0.78 (0.35, 1.77)	0.56	0.70 (0.29, 1.67)	0.56
**Marital status**	Married	1.00 (ref)	-	1.00 (ref)	-
	Widowed	0.59 (0.15, 2.35)	0.46	0.77 (0.19, 3.15)	0.46
	Divorced/Separated	1.48 (0.8, 2.75)	0.21	1.29 (0.65, 2.55)	0.21
	Never married	0.95 (0.41, 2.16)	0.89	0.77 (0.30, 2.01)	0.89
**Time incarcerated**	≤1 month	4.00 (0.99, 16.16)	0.05	4.54 (0.98, 21.11)	0.05
	>1-6 months	2.30 (0.71, 7.51)	0.17	2.45 (0.80, 7.54)	0.17
	>6-24 months	3.03 (0.98, 9.42)	0.06	3.06 (1.03, 9.11)	0.06
	>24 months	1.00 (ref)	-	1.00 (ref)	-
**Pre-incarceration alcohol use[c]**	No unhealthy alcohol use	1.00 (ref)	-	1.00 (ref)	-
	Unhealthy alcohol use	0.46 (0.17, 1.23)	0.12	0.39 (0.15, 1.01)	0.12
	Missing	1.23 (0.58, 2.58)	0.59	1.32 (0.64, 2.73)	0.59

Note: RR- risk ratio, aRR- adjusted risk ratio, CI- confidence interval; ref- reference
[a]As assessed by verbal interview using the AUDIT.
[b]As assessed by verbal interview using the DUDIT.
[c]As assessed by verbal interview using the AUDIT-C.
Adjustment set includes age, sex, marital status, time incarcerated, pre-release alcohol use, and time to viral load measurement at follow-up (as random effect).

© 2025 Herce ME et al. *JAMA Network Open*.

eTable 3a. Inverse probability weighted risk ratios for loss of viral suppression among participants with vs without unhealthy alcohol use who were virally suppressed pre-release (N=205).

Factor	Level	aRR	95% CI	p-value
**Unhealthy alcohol use[a]**	No	1.00 (ref)	-	-
	Yes	3.95	(1.12, 13.90)	0.03
**Age**	18-24 years	5.63	(0.23, 138.38)	0.29
	25-29 years	14.45	(2.72, 76.89)	0.002
	30-34 years	9.61	(1.76, 52.34)	0.009
	35-39 years	1.00 (ref)	-	-
	40-44 years	2.44	(0.36, 16.71)	0.36
	45+ years	0.65	(0.07, 5.73)	0.70
**Birth sex**	Male	1.00 (ref)	-	-
	Female	1.33	(0.35, 5.07)	0.67
**Marital status**	Married	1.00 (ref)	-	-
	Widowed	2.50	(0.56, 11.10)	0.23
	Divorced/Separated	0.94	(0.21, 4.12)	0.93
	Never married	0.28	(0.03, 2.44)	0.25
	Unknown	n/a	n/a	n/a
**Time incarcerated**	≤1 month	5.30	(0.58, 48.65)	0.14
	>1-6 months	3.03	(0.60, 15.48)	0.18
	>6-24 months	1.67	(0.25, 11.21)	0.60
	>24 months	1.00 (ref)	-	-
**Pre-incarceration alcohol use[b]**	No unhealthy alcohol use	1.00 (ref)	-	-
	Unhealthy alcohol use	0.35	(0.09, 1.35)	0.13
	Missing	1.03	(0.14, 7.66)	0.98

Note: aRR- adjusted risk ratio; CI- confidence interval; ref- reference
[a]As assessed by verbal interview using the AUDIT.
[b]As assessed by verbal interview using the AUDIT-C.
Adjusted risk ratio inverse probability weighted for unhealthy alcohol use, with analysis among those virally suppressed at release (viral suppression defined as <1,000 copies/mL).
Time incarcerated measured at release date.
Adjustment set includes age, birth sex, marital status, time incarcerated, pre-incarceration alcohol use, and time to viral load measurement at follow-up (as random effect); n/a - insufficient data to calculate estimate.

© 2025 Herce ME et al. *JAMA Network Open*.

eTable 3b. Inverse probability weighted risk ratios for loss of viral suppression among participants with vs without unhealthy drug use who were virally suppressed pre-release (N=209).

Factor	Level	aRR	95% CI	p-value
**Unhealthy drug use[a]**	No	1.00 (ref)	-	-
	Yes	5.67	(1.72, 18.68)	0.004
**Age**	18-24 years	4.38	(0.16, 116.57)	0.38
	25-29 years	11.37	(2.23, 58.02)	0.003
	30-34 years	11.32	(2.22, 57.75)	0.004
	35-39 years	1.00 (ref)	-	-
	40-44 years	3.44	(0.57, 20.68)	0.18
	45+ years	0.82	(0.10, 6.97)	0.86
**Birth sex**	Male	1.00 (ref)	-	-
	Female	1.66	(0.45, 6.10)	0.45
**Marital status**	Married	1.00 (ref)	-	-
	Widowed	2.10	(0.50, 8.75)	0.31
	Divorced/Separated	0.74	(0.15, 3.77)	0.72
	Never married	0.37	(0.05, 2.56)	0.31
	Unknown	n/a	n/a	n/a
**Time incarcerated**	≤1 month	6.84	(0.73, 64.18)	0.09
	>1-6 months	2.78	(0.47, 16.41)	0.26
	>6-24 months	1.99	(0.30, 13.29)	0.48
	>24 months	1.00 (ref)	-	-
**Pre-incarceration alcohol use[b]**	No unhealthy alcohol use	1.00 (ref)	-	-
	Unhealthy alcohol use	0.31	(0.07, 1.31)	0.11
	Missing	1.16	(0.21, 6.44)	0.86

Note: aRR- adjusted risk ratio; CI- confidence interval; ref- reference
[a]As assessed by verbal interview using the DUDIT**.**
[b]As assessed by verbal interview using the AUDIT-C.
Adjusted risk ratio inverse probability weighted for unhealthy drug use, with analysis among those virally suppressed at release (viral suppression defined as <1,000 copies/mL).
Time incarcerated measured at release date.
Adjustment set includes age, birth sex, marital status, time incarcerated, pre-incarceration alcohol use, and time to viral load measurement at follow-up (as random effect); n/a - insufficient data to calculate estimate.

© 2025 Herce ME et al. *JAMA Network Open*.

eFigure 1. Directed acyclic graph (DAG), featuring minimal adjustment set of age, marital status, sex, pre-incarceration alcohol use, and time incarcerated.

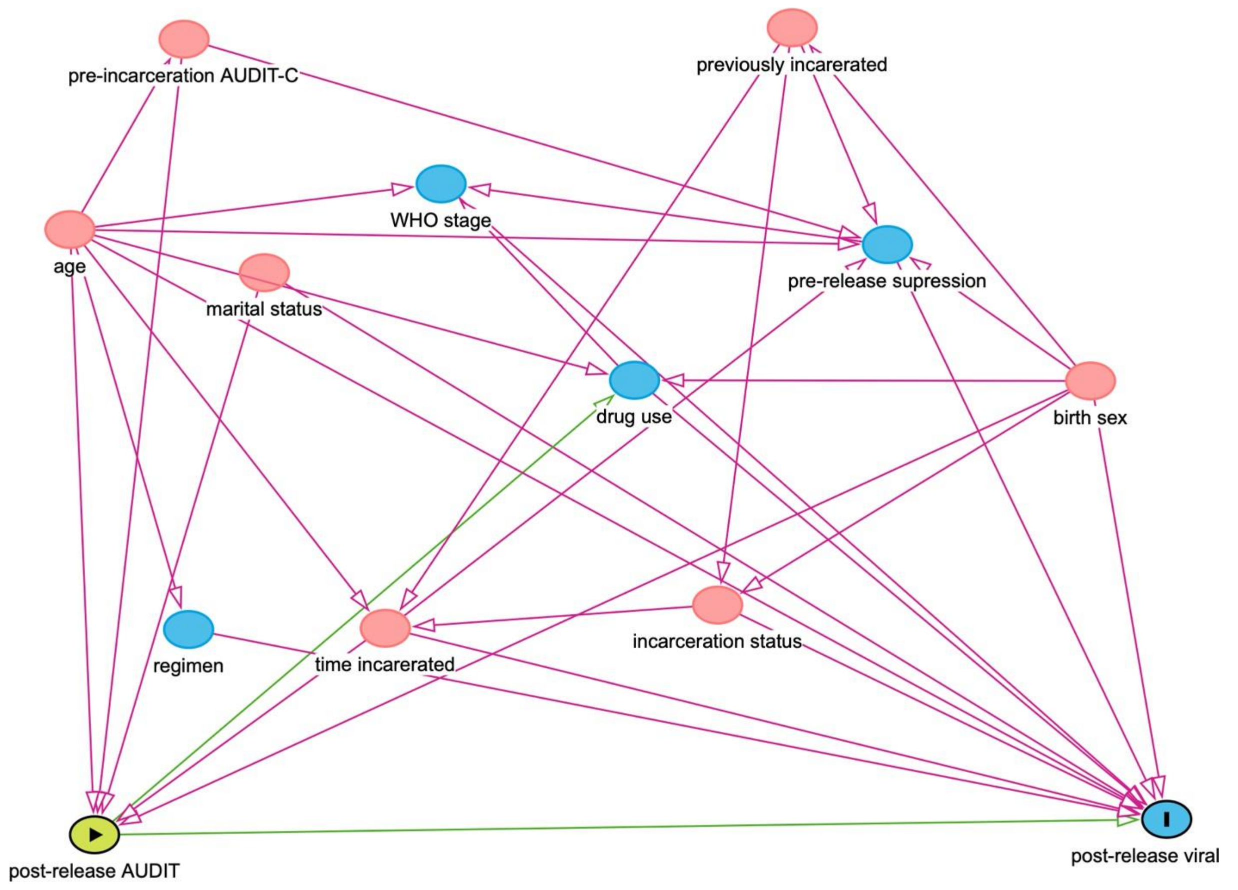

Back-door path(s)/Adjustment sets considered:

- age, birth sex, marital status, pre-incarceration AUDIT-C, time incarcerated
- age, birth sex, marital status, pre-release suppression, previously incarcerated, time incarcerated
- age, birth sex, incarceration status, marital status, pre-release suppression, time incarcerated

Conditional independences:

- WHO stage ⊥ birth sex | age, drug use, pre-release suppression
- WHO stage ⊥ incarceration status | age, birth sex, previously incarcerated, time incarcerated
- WHO stage ⊥ incarceration status | age, birth sex, pre-incarceration AUDIT-C, pre-release suppression, time incarcerated
- WHO stage ⊥ incarceration status | age, birth sex, post-release AUDIT, pre-release suppression
- WHO stage ⊥ incarceration status | age, drug use, pre-release suppression
- WHO stage ⊥ marital status | age, birth sex, post-release AUDIT, pre-incarceration AUDIT-C, time incarcerated
- WHO stage ⊥ marital status | age, birth sex, post-release AUDIT, pre-release suppression
- WHO stage ⊥ marital status | age, birth sex, drug use, pre-incarceration AUDIT-C, time incarcerated
- WHO stage ⊥ marital status | age, drug use, pre-release suppression
- WHO stage ⊥ post-release AUDIT | age, birth sex, drug use, pre-incarceration AUDIT-C, time incarcerated
- WHO stage ⊥ post-release AUDIT | age, drug use, pre-release suppression
- WHO stage ⊥ pre-incarceration AUDIT-C | age, birth sex, post-release AUDIT, pre-release suppression

© 2025 Herce ME et al. *JAMA Network Open*.

- WHO stage ⊥ pre-incarceration AUDIT-C | age, drug use, pre-release suppression
- WHO stage ⊥ previously incarcerated | age, birth sex, pre-incarceration AUDIT-C, pre-release suppression, time incarcerated
- WHO stage ⊥ previously incarcerated | age, birth sex, post-release AUDIT, pre-release suppression
- WHO stage ⊥ previously incarcerated | age, drug use, pre-release suppression
- WHO stage ⊥ time incarcerated | age, birth sex, post-release AUDIT, pre-release suppression
- WHO stage ⊥ time incarcerated | age, drug use, pre-release suppression
- WHO stage ⊥ regimen | age
- birth sex ⊥ marital status
- birth sex ⊥ pre-incarceration AUDIT-C
- birth sex ⊥ time incarcerated | incarceration status, previously incarcerated
- birth sex ⊥ age
- birth sex ⊥ regimen
- drug use ⊥ incarceration status | age, birth sex, time incarcerated
- drug use ⊥ incarceration status | age, birth sex, post-release AUDIT
- drug use ⊥ marital status | age, birth sex, post-release AUDIT
- drug use ⊥ pre-incarceration AUDIT-C | age, birth sex, post-release AUDIT
- drug use ⊥ pre-release suppression | age, birth sex, pre-incarceration AUDIT-C, time incarcerated
- drug use ⊥ pre-release suppression | age, birth sex, post-release AUDIT
- drug use ⊥ previously incarcerated | age, birth sex, time incarcerated
- drug use ⊥ previously incarcerated | age, birth sex, post-release AUDIT
- drug use ⊥ time incarcerated | age, birth sex, post-release AUDIT
- drug use ⊥ regimen | age
- incarceration status ⊥ marital status
- incarceration status ⊥ post-release AUDIT | age, birth sex, time incarcerated
- incarceration status ⊥ pre-incarceration AUDIT-C
- incarceration status ⊥ pre-release suppression | age, birth sex, previously incarcerated, time incarcerated
- incarceration status ⊥ age
- incarceration status ⊥ regimen
- marital status ⊥ pre-incarceration AUDIT-C
- marital status ⊥ pre-release suppression
- marital status ⊥ previously incarcerated
- marital status ⊥ time incarcerated
- marital status ⊥ age
- marital status ⊥ regimen
- post-release AUDIT ⊥ pre-release suppression | age, birth sex, pre-incarceration AUDIT-C, time incarcerated
- post-release AUDIT ⊥ previously incarcerated | age, birth sex, time incarcerated
- post-release AUDIT ⊥ regimen | age
- post-release viral load ⊥ pre-incarceration AUDIT-C | age, birth sex, marital status, post-release AUDIT, pre-release suppression, previously incarcerated, time incarcerated
- post-release viral load ⊥ pre-incarceration AUDIT-C | age, birth sex, incarceration status, marital status, post-release AUDIT, pre-release suppression, time incarcerated
- post-release viral load ⊥ previously incarcerated | age, birth sex, incarceration status, pre-incarceration AUDIT-C, pre-release suppression, time incarcerated
- post-release viral load ⊥ previously incarcerated | age, birth sex, incarceration status, marital status, post-release AUDIT, pre-release suppression, time incarcerated
- pre-incarceration AUDIT-C ⊥ previously incarcerated
- pre-incarceration AUDIT-C ⊥ time incarcerated | age
- pre-incarceration AUDIT-C ⊥ regimen | age
- pre-release suppression ⊥ regimen | age
- previously incarcerated ⊥ age
- previously incarcerated ⊥ regimen
- time incarcerated ⊥ regimen | age

© 2025 Herce ME et al. *JAMA Network Open*.

eFigure 2. Histogram of time from release to study follow-up completion (N=251).

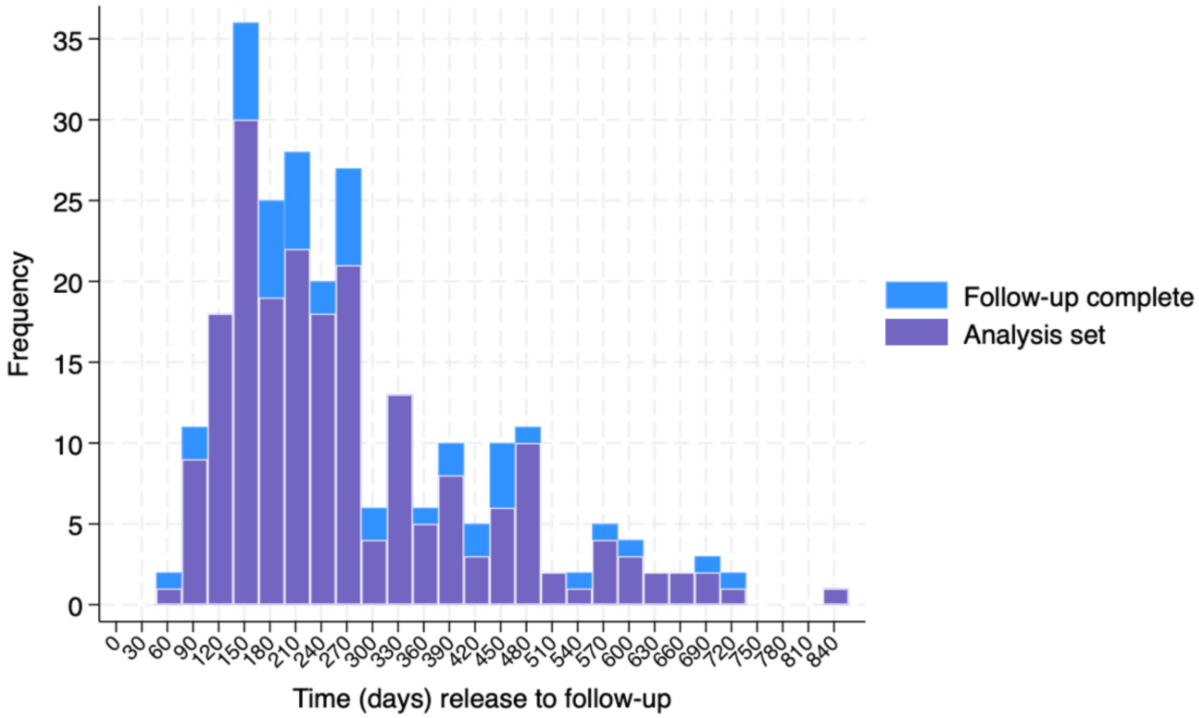

© 2025 Herce ME et al. *JAMA Network Open*.
