# Supplementary material for: Alcohol Use and HIV Suppression After Release From Prison Among People With HIV in Zambia
Source: JAMA Netw Open. 2025 Dec 5;8(12):e2547295. doi: 10.1001/jamanetworkopen.2025.47295 (PMC12681033; doi:10.1001/jamanetworkopen.2025.47295)
Supplement: Supplement 2. — Data Sharing Statement [file jamanetwopen-e2547295-s002.pdf]

Data Sharing Statement

Herce. Alcohol Use and HIV Suppression After Release From Prison Among People With HIV in Zambia. *JAMA Netw Open*. Published December 05, 2025.
doi:10.1001/jamanetworkopen.2025.47295

Data
Data available: Yes
Data types: Other (please specify)
Additional Information: Deidentified participant database
How to access data: The de-identified cohort database and data dictionary will be made available within 30 days of publication. The analytical dataset includes routine de-identified medical record data that are the property of the Government of Zambia, and, as such, use of these data requires local permission when applicable conditions are met. To request permission to access these data, please submit a request in writing via email to the study PI, Michael Herce, at michael.herce@cidrz.org.
When available: With publication

Supporting Documents
Document types: Other (please specify)
Additional Information: Data dictionary
How to access documents: The de-identified cohort database and data dictionary will be made available within 30 days of publication. The analytical dataset includes routine de-identified medical record data that are the property of the Government of Zambia, and, as such, use of these data requires local permission when applicable conditions are met. To request permission to access these data, please submit a request in writing via email to the study PI, Michael Herce, at michael.herce@cidrz.org.
When available: With publication

Additional Information
Who can access the data: The de-identified cohort database and data dictionary will be made available within 30 days of publication. The analytical dataset includes routine de-identified medical record data that are the property of the Government of Zambia, and, as such, use of these data requires local permission when applicable conditions are met. To request permission to access these data, please submit a request in writing via email to the study PI, Michael Herce, at michael.herce@cidrz.org.
Types of analyses: The analyses can be used for any purpose that does not involve attempts at individual participant identity deduction.
Mechanisms of data availability: The database and data dictionary will be made available after a signed data access agreement is completed with the requesting party and the Centre for Infectious Disease Research in Zambia (CIDRZ) specifying the use of the data and secure methods for data transfer.
